# TRPM4 modulates endothelial inflammation and pyroptosis via the HSP60-NF-κB axis

**DOI:** 10.3389/fphar.2026.1823348

**Published:** 2026-04-24

**Authors:** Meimei Shen, Yu Zhao, Yuyao Zhang, Tingting Tong, Yunfeng Cui, Xin Guo, Wen Liang, Ziyue Ma, Jing Jin, Lisi Xiong, Ke Tang, Kaiyang Gao, Junhao Zhang, Hongzhao Lv, Rong Huo, Tao Ban

**Affiliations:** 1 Department of Pharmacology (The Key Laboratory of Cardiovascular Research, Ministry of Education, State Key Laboratory of Frigid Zone Cardiovascular Diseases, Ministry of Science and Technology) at College of Pharmacy, Harbin Medical University, Harbin, China; 2 Department of Anatomy, School of Basic Medical Sciences, Heilongjiang University of Chinese Medicine, Harbin, China; 3 Clinical Pharmacy Office, Department of Pharmacy, The Second People’s Hospital of Heihe, Heihe, China; 4 Department of General Surgery, The Fourth Affiliated Hospital of Harbin Medical University, Harbin, China; 5 Heilongjiang Academy of Medical Sciences, Harbin, China; 6 National-Local Joint Engineering Laboratory of Drug Research and Development of Cardio-Cerebrovascular Diseases in Frigid Zone, The National Development and Reform Commission, Harbin, China

**Keywords:** endothelial inflammation, HSP60, NF-κB signaling pathway, pyroptosis, TRPM4

## Abstract

**Background:**

Atherosclerosis is a chronic inflammatory condition of the arterial wall in which endothelial dysfunction serves as a key driver of disease progression. Endothelial inflammation and pyroptosis are major contributors in this context; therefore, targeting these processes may confer therapeutic benefits. Transient receptor potential cation channel subfamily M member 4 (TRPM4) is a voltage-sensitive, non-selective cation channel belonging to the transient receptor potential family. Although TRPM4 contributes to the regulation of vascular endothelium, its precise role in endothelial inflammation remains poorly understood. Accordingly, this study aims to elucidate the function and molecular mechanisms of TRPM4 in vascular endothelial inflammation and pyroptosis.

**Methods:**

An *in vitro* model of endothelial inflammation and pyroptosis was established by stimulating human umbilical vein endothelial cells (HUVECs) with tumor necrosis factor-α (TNF-α). The TRPM4-specific inhibitor 9-Phenanthrol (9-Phe) was applied to assess TRPM4 involvement. Expression levels of TRPM4, inflammatory adhesion molecules (VCAM-1, ICAM-1), pyroptosis-related proteins (NLRP3, cleaved caspase-1, GSDMD, IL-1β, IL-18), and key transcriptional regulators were quantified via quantitative real-time PCR (qRT-PCR) and Western blot. Intracellular calcium flux was measured using Fluo-4 AM. We used co-immunoprecipitation to assess the interaction between HSP60 and the IKKα/β complex, and Immunofluorescence to visualize nuclear translocation of phosphorylated NF-κB p65.

**Results:**

TNF-α stimulation significantly upregulated the expression of TRPM4. Administration of the TRPM4 inhibitor 9-Phe attenuated this increase. Treatment with 9-Phe also reduced the TNF-α-induced elevation of adhesion molecules VCAM-1 and ICAM-1. It further decreased the expression of pyroptosis-related markers, including NLRP3, caspase-1, GSDMD, IL-1β, and IL-18. In addition, 9-Phe markedly reduced the TNF-α-driven nuclear translocation of phosphorylated NF-κB p65. HSP60 knockdown intensified TNF-α-induced inflammation and pyroptosis. Mechanistic analysis showed that TRPM4 inhibition reduced the interaction between HSP60 and the IKKα/β complex.

**Conclusion:**

TRPM4 plays a critical role in TNF-α-induced endothelial inflammation and pyroptosis, and its inhibition attenuates these pathological changes. Mechanistic findings indicate that TRPM4 promotes the interaction between HSP60 and IKKα/β, thereby activating the NF-κB pathway. Collectively, these results identify the TRPM4-HSP60-NF-κB axis as a central regulator of endothelial inflammation and pyroptosis, and suggest its potential as a therapeutic target for atherosclerosis.

## 1 Introduction

The vascular endothelium acts as a key interface between blood and tissues, ensuring vascular homeostasis and protecting against pathological damage (Xu S. et al., 2021). Endothelial dysfunction marks the early phase of atherosclerosis. Chronic inflammation and pyroptosis in endothelial cells contribute to disease progression (Bu et al., 2023). Caspase-1 activation in endothelial cells induces a pro-inflammatory state and enhances monocyte recruitment. These effects contribute to the progression of atherosclerosis (Yao et al., 2022; Sharma and Kanneganti, 2021; Jiang et al., 2023). Inhibition of pyroptosis has been shown to reduce atherosclerosis (Wei et al., 2023); therefore, targeting pyroptosis represents a promising therapeutic strategy.

TRPM4 is a calcium-activated, voltage-dependent, non-selective cation channel. It is expressed in various tissues, including the cardiovascular system, where it helps regulate endothelial cell function (Cheng et al., 2007). Recent studies show that TRPM4 contributes to vascular inflammation (Becerra et al., 2011). Our previous work has demonstrated that pyroptosis plays a critical role in various types of endothelial injury. This finding closely associates TRPM4 with endothelial dysfunction (Ding et al., 2017). However, how TRPM4 regulates endothelial inflammation and pyroptosis is still not fully understood.

Heat shock protein 60 (HSP60) is a molecular chaperone. Pyroptosis is involved in various cellular processes and is closely associated with atherosclerosis (Zonnar et al., 2022; Knoflach et al., 2005; Ayada et al., 2009). Inhibiting HSP60 slows the progression of atherosclerosis and reduces NLRP3 inflammasome activation (Khwaja et al., 2021). This suggests a potential connection to pyroptosis signaling. Despite the established association of both TRPM4 and HSP60 with endothelial pathology, whether TRPM4 modulates endothelial inflammation and pyroptosis through HSP60 remains unknown. This study aimed to define the specific role of TRPM4 in vascular endothelial inflammation and pyroptosis. We also aimed to explore whether TRPM4 interacts molecularly with the HSP60 signaling pathway.

## 2 Materials and methods

### 2.1 Cell culture

Human umbilical vein endothelial cells (HUVECs) were obtained from the Department of Pharmacology at Harbin Medical University (Harbin, China). The cells were cultured in RPMI-1640 medium (C11875500BT, United States) supplemented with 10% fetal bovine serum (04-010-1A, BI, Israel) and 1% antibiotic-antimycotic solution (P7630, Solarbio, China). Cultures were maintained at 37 °C in a humidified atmosphere with 5% CO_2_.

### 2.2 Western blot analysis

HUVECs were cultured in 6-well plates. Following treatment, the medium was discarded, and the cells were washed three times with ice-cold PBS. Residual buffer was thoroughly aspirated. Cells were placed on ice and lysed with RIPA buffer supplemented with protease and phosphatase inhibitors. The lysates were collected and subjected to intermittent ultrasonication for 15–20 s, followed by centrifugation at 13,500 rpm for 15 min at 4 °C. The resulting supernatant was collected for protein quantification and immunoblot analysis.

Protein concentrations were determined using a BCA Protein Assay Kit (P0012, Beyotime, China). Equal amounts of protein (80 μg per well) were loaded onto an SDS-PAGE gel for electrophoretic separation. The separated proteins were then transferred onto a nitrocellulose membrane (PALL Gelman, USA). The membrane was blocked with 5% skim milk at room temperature for 1 h, followed by overnight incubation with the specific primary antibody at 4 °C. After washing, the membrane was incubated with the appropriate fluorescent secondary antibody (1:7500, Abbkine, USA) at room temperature for 1 h in the dark. Protein bands were visualized and quantified using the Odyssey infrared imaging system (LI-COR Biosciences, USA).

### 2.3 Co-immunoprecipitation (Co-IP)

Cell lysates were prepared from HUVECs as described in Section 2.2. For co-immunoprecipitation, 300 μg of total protein was incubated with the target primary antibody overnight at 4 °C with gentle rotation. Subsequently, 40 μL of Protein A/G magnetic beads were added, and the mixture was incubated with continuous shaking overnight at 4 °C. The bead–antibody–antigen complex was then thoroughly washed with PBST (PBS containing 0.25% Tween-20). After the final wash, the tubes were placed on a magnetic stand to collect the beads. The captured complexes were then mixed with RIPA lysis buffer and SDS-PAGE loading buffer for subsequent Western blot analysis.

### 2.4 Quantitative real-time PCR (qRT-PCR)

HUVECs were cultured in 6-well plates. Total RNA was extracted using TRIZOL reagent (15596026CN, Invitrogen, USA) according to the manufacturer’s instructions. Equal amounts of RNA were used for reverse transcription, and cDNA was synthesized using the ReverTra Ace qPCR RT Master Mix kit (FSQ-201, TOYOBO, Japan). Quantitative PCR was performed on an ABI QS6 system using FastStart Universal SYBR Green Master Mix (Rox; Roche, Switzerland) and gene-specific primers. The primer sequences used are listed in Table 1. The mRNA expression levels of the target gene were normalized to the endogenous reference genes GAPDH, Relative quantification of gene expression was calculated using the comparative threshold cycle (2^−ΔΔCT^) method.

**TABLE 1:** qRT-PCR primer sequence.

Name	Sequence of forward primer(F)	Sequence of reverse primer(R)
Human TRPM4	GCA​CGA​CGT​TCA​TAG​TTG​ACT	CTT​CTC​CGT​GGT​GTG​TGC​AT
Human HSP60	CCA​CTG​CTA​CTG​TAC​TGG​CAC	AGC​TAA​CAT​CAC​ACC​TCT​CCT
Human IL-6	ACT​CAC​CTC​TTC​AGA​ACG​AAT​TG	CCA​TCT​TTG​GAA​GGT​TCA​GGT​TG
Human IL-8	TTT​TGC​CAA​GGA​GTG​CTA​AAG​A	AAC​CCT​CTG​CAC​CCA​GTT​TTC
Human VCAM-1	GGG​AAG​ATG​GTC​GTG​ATC​CTT	TCT​GGG​GTG​GTC​TCG​ATT​TTA
Human ICAM-1	ATG​CCC​AGA​CAT​CTG​TGT​CC	GGG​GTC​TCT​ATG​CCC​AAC​AA
Human IL-1β	TTC​GAC​ACA​TGG​GAT​AAC​GAG​G	TTT​TTG​CTG​TGA​GTC​CCG​GAG
Human NLRP3	CAC​CTG​TTG​TGC​AAT​CTG​AAG	GCA​AGA​TCC​TGA​CAA​CAT​GC
Human Caspase-1	CCT​TAA​TAT​GCA​AGA​CTC​TCA​AGG​A	TAA​GCT​GGG​TTG​TCC​TGC​ACT

### 2.5 Cell transfection

Small interfering RNA (siRNA) targeting HSP60 was used to achieve gene silencing, and a non-targeting scrambled siRNA was applied as a negative control. For transfection in 6-well plates, 50 nM siRNA and 5 μL Lipofectamine 2000 (Invitrogen, USA) were diluted separately in antibiotic-free DMEM, gently mixed, and then incubated with the cells at 37 °C for 6 h. Following transfection, the medium was replaced with fresh complete culture medium containing serum and antibiotics.

### 2.6 Determination of intracellular calcium ion concentration

The fluorescent calcium indicator Fluo-4 AM (S1060, Beyotime, China) was used to monitor intracellular calcium levels. HUVECs were seeded onto culture slides placed in 12-well plates and allowed to adhere for 24 h at 37 °C. The cells were then loaded with 2 μM Fluo-4 AM solution and incubated for 30 min at 37 °C in the dark. Following loading, fluorescence images were captured using a confocal microscope (excitation 488 nm), and emission signals were quantified to evaluate relative changes in cytosolic calcium concentration.

### 2.7 Immunofluorescence

HUVECs were seeded onto glass coverslips in 12-well plates and allowed to attach. Following the designated transfection or drug treatment, cells were washed with PBS and fixed with 4% paraformaldehyde for 15 min at room temperature. Cell membranes were permeabilized with 0.1% Triton X-100 for 30 min, and non-specific binding sites were blocked with 10% goat serum for 1 h at room temperature. Subsequently, cells were incubated overnight at 4 °C with a primary antibody against Phospho-NF-κB p65 (Ser536) (ABclonal, China) diluted 1:100. The following day, cells were incubated with an Alexa Fluor-conjugated secondary antibody (1:200 dilution, Beyotime, China) for 1 h at room temperature in the dark. Nuclei were counterstained with DAPI for 10 min. Fluorescence images were acquired using a confocal laser scanning microscope (Carl Zeiss LSM 800, Germany) and analyzed with appropriate image processing software.

### 2.8 Statistical analysis

All quantitative data are presented as mean ± standard deviation (SD) from at least three independent experiments. Statistical comparisons between two groups were performed using an unpaired, two-tailed Student’s t-test. Multiple group comparisons were conducted using one-way analysis of variance (ANOVA) followed by appropriate *post hoc* tests (such as Tukey or Bonferroni tests) using GraphPad Prism software. A p-value of less than 0.05 was considered statistically significant.

## 3 Results

### 3.1 TNF-α upregulates TRPM4 expression in endothelial inflammation

**FIGURE 1:**
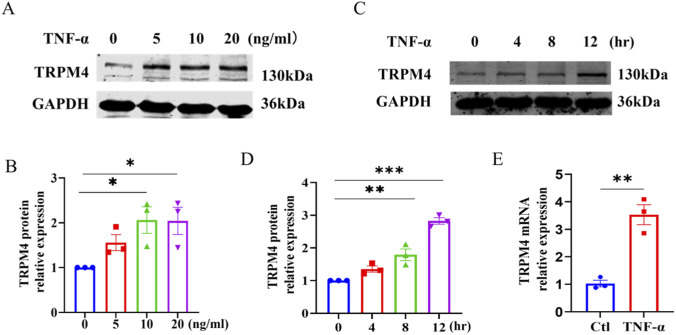
TNF-α upregulates TRPM4 expression in HUVECs. **(A,B)** HUVECs were treated with increasing concentrations of TNF-α for 12 h, TRPM4 protein expression was analyzed by Western blot. *P < 0.05 vs. 0 ng/mL; n = 3. **(C,D)** HUVECs were treated with 20 ng/mL TNF-α for the indicated durations (0, 4, 8, 12 h), TRPM4 protein levels were assessed by Western blot. **P < 0.01, ***P < 0.001 vs. 0 h; n = 3. **(E)** HUVECs were treated with 20 ng/mL of TNF-α for 12 h, the mRNA expression of TRPM4 was quantified by qRT-PCR. **P < 0.01 vs. ctl; n = 3.

Endothelial inflammation and dysfunction are established initiating factors in the pathogenesis of AS. Tumor necrosis factor-α (TNF-α) is a key pro-inflammatory cytokine that drives immune activation and various forms of programmed cell death. It is widely used to establish *in vitro* models of endothelial inflammation and injury relevant to atherosclerosis (Zhao et al., 2023). To investigate the potential role of TRPM4 in this context, we stimulated HUVECs with TNF-α. The results demonstrated that TNF-α treatment increased TRPM4 protein expression in a dose- and time-dependent manner, with the most pronounced upregulation observed at a concentration of 20 ng/mL (Figures 1A–D). This concentration was therefore selected for subsequent experiments. Consistent with the protein-level findings, qRT-PCR analysis revealed a significant increase in TRPM4 mRNA expression following TNF-α stimulation (Figure 1E). These findings suggest that TRPM4 may play an important role in TNF-α-induced endothelial inflammation.

### 3.2 Pharmacological inhibition of TRPM4 attenuates TNF-α-induced endothelial inflammation

**FIGURE 2:**
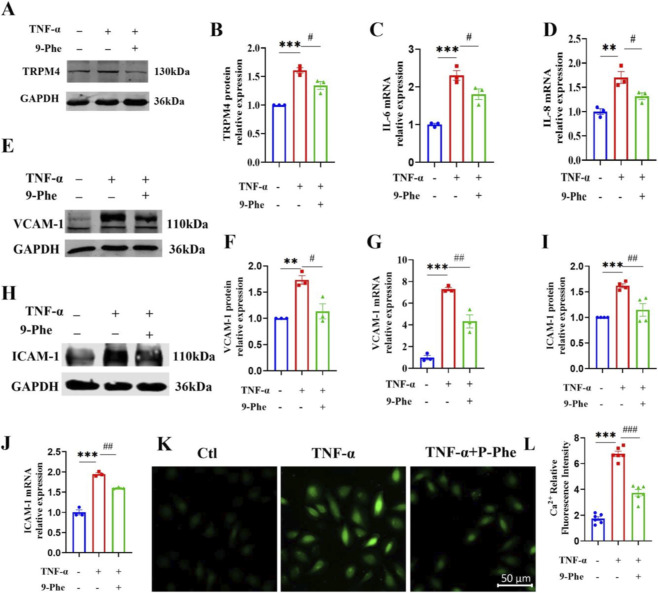
Inhibition of TRPM4 attenuates TNF-α-induced inflammatory response in HUVECs. **(A,B)** HUVECs were treated with 20 ng/mL of TNF-α in the presence or absence of 1 μM of 9-Phe for 12 h. Western blot analysis of TRPM4 protein expression. ***P < 0.001 vs. Ctl; ^#^P < 0.05 vs. TNF-α; n = 3. **(C,D)** The mRNA expressions of IL-6 and IL-8 were determined by qRT-PCR. ***P < 0.001, **P < 0.01 vs. Ctl; ^#^P < 0.05 vs. TNF-α; n = 3. **(E–G)** Protein expression levels of VCAM-1 were assessed by Western blot and the mRNA expressions of VCAM-1 was determined by qRT-PCR. ***P < 0.001 vs. Ctl; **P < 0.01 vs. Ctl; ^##^P < 0.01 vs. TNF-α; ^#^P < 0.05 vs. TNF-α; n = 3. **(H–J)** Protein expression levels of ICAM-1 were assessed by Western blot and the mRNA expressions of ICAM-1 was determined by qRT-PCR. ***P < 0.001 vs. Ctl; ^##^P < 0.01 vs. TNF-α; n = 3. **(G–J)** Protein expression levels of VCAM-1 and ICAM-1 were assessed by Western blot. ***P < 0.001, **P < 0.01 vs. Ctl; ^##^P < 0.01, ^#^P < 0.05 vs. TNF-α; n = 3–4. **(K)** Representative fluorescence images of HUVECs stained with Fluo-4 AM. **(L)** Quantitative analysis of Fluo-4 AM fluorescence intensity. ***P < 0.001 vs. Ctl; ^###^P < 0.001 vs. TNF-α; n = 6.

Inflammatory activation of endothelial cells induces a sustained pro-inflammatory phenotype, characterized by upregulated expression of adhesion molecules (including VCAM-1 and ICAM-1) and increased secretion of chemokines (including MCP-1 and IL-8) (Xu S. et al., 2021). To examine the role of TRPM4 in this process, HUVECs were exposed to TNF-α with or without the TRPM4-specific inhibitor 9-Phenanthrol (9-Phe). Treatment with 9-Phe attenuated the TNF-α-induced upregulation of TRPM4 protein expression (Figures 2A,B). It should be noted that 9-Phe is primarily characterized as a functional inhibitor of the TRPM4 channel, therefore, its effect on TRPM4 protein levels may involve feedback regulation or an indirect effect on protein stability (Guinamard et al., 2014). Furthermore, 9-Phe significantly reduced the mRNA and protein levels of TNF-α-induced inflammatory mediators, specifically IL-6, IL-8, VCAM-1, and ICAM-1 (Figures 2C–J). Given the critical role of these adhesion molecules in mediating monocyte recruitment and foam cell formation, their suppression suggests a mechanistic basis by which TRPM4 inhibition may attenuate early atherogenic events. We previously reported that intracellular calcium concentration was elevated during palmitic acid-induced endothelial injury, an effect partially reversed by TRPM4 silencing (Ye et al., 2024). Here, we examined whether calcium signaling also contributes to TNF-α-induced inflammation. Consistent with a role for TRPM4 in calcium homeostasis, TNF-α increased Fluo-4 AM fluorescence intensity in HUVECs, indicating elevated cytosolic calcium, this increase was substantially attenuated by 9-Phe (Figures 2K,L). These results suggest that TRPM4 in the modulation of intracellular calcium dynamics during inflammation, which may be involved in endothelial activation.

### 3.3 Pharmacological inhibition of TRPM4 attenuates TNF-α-induced endothelial pyroptosis

**FIGURE 3:**
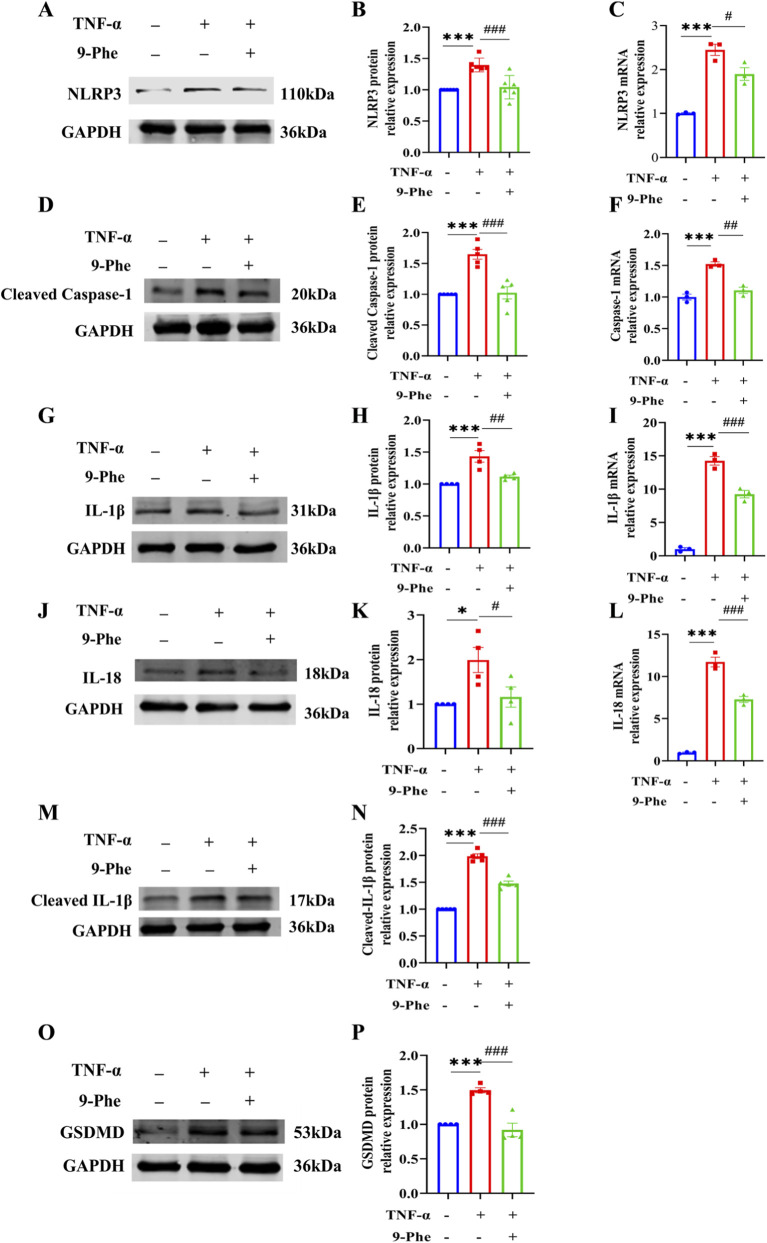
TRPM4 inhibition attenuates TNF-α-induced pyroptosis in HUVECs. **(A–P)** The mRNA expressions of NLRP3, Caspase-1; IL-1β and IL-18 were determined by qRT-PCR and protein expression levels of NLRP3, GSDMD, Cleaved Caspase-1, IL-18, IL-1β, and Cleaved IL-1β were analyzed by Western blot. ***P < 0.001, *P < 0.001 vs. ctl; ^###^P < 0.0001, ^##^P < 0.01, ^#^P < 0.05 vs. TNF-α; n = 4-6.

Given that sustained inflammatory stress often leads to programmed cell death (Shi et al., 2017; Yu et al., 2021), we next examined whether TNF-α induced pyroptosis-a lytic form of cell death driven by caspase-1 activation that releases pro-inflammatory cytokines and amplifies inflammatory responses (Miao et al., 2023; Li et al., 2021; Zeng et al., 2021). mRNA levels of the pyroptosis-related genes-NLRP3, caspase-1, IL-1β, and IL-18-were upregulated following TNF-α treatment, and protein levels were correspondingly elevated. TNF-α upregulated the protein levels of NLRP3, GSDMD, cleaved caspase-1, IL-18, IL-1β, and cleaved IL-1β in HUVECs, indicative of pyroptosis induction. Conversely, treatment with 9-Phe, a TRPM4 inhibitor, reduced the levels of these proteins. Treatment with 9-Phe reduced both the mRNA and protein levels of these pyroptosis markers (Figures 3A–P). These findings indicate that TRPM4 plays a critical role in mediating endothelial pyroptosis and inflammasome activation, contributing to the mechanisms by which TNF-α induces endothelial inflammation.

### 3.4 TRPM4 mediates TNF-α induced endothelial inflammation and pyroptosis via HSP60

**FIGURE 4:**
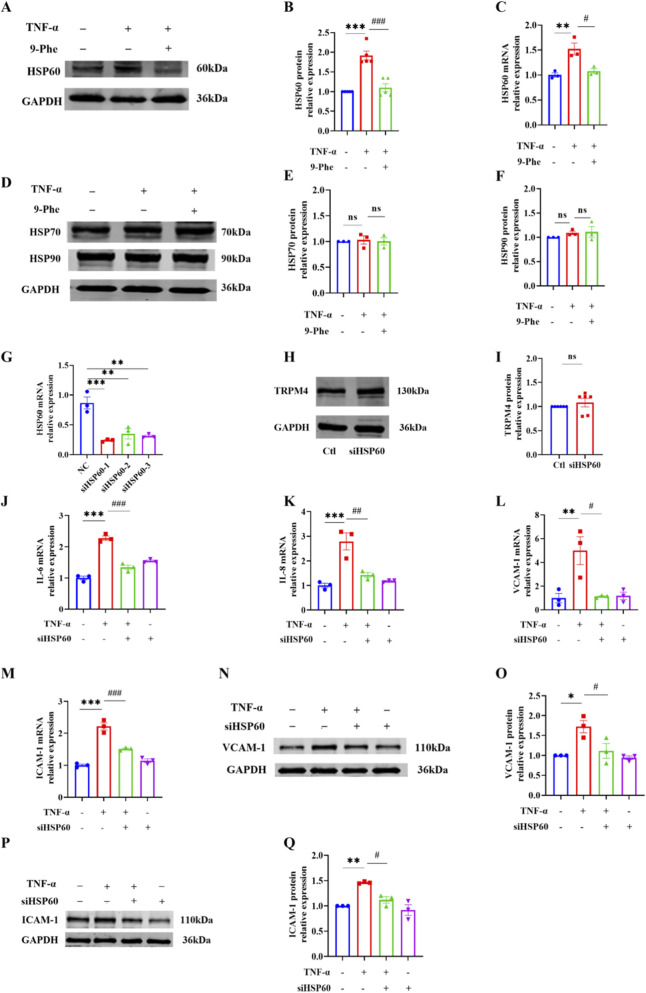
TRPM4 inhibition attenuates TNF-α-induced inflammatory response via HSP60 regulation. **(A,B)** HUVECs were treated with 20 ng/mL of TNF-α in the presence or absence of 1 μM of 9-Phe for 12 h. Protein expression of HSP60 was analyzed by Western blot. ***P < 0.001 vs. Ctl; ^###^P < 0.001 vs. TNF-α; n = 5. **(C)** The mRNA expression of HSP60 was determined by qRT-PCR. **P < 0.01 vs. Ctl, ^#^P < 0.05 vs. TNF-α; n = 3. **(D–F)** The protein expression of HSP90 and HSP70 were determined by Western blot. Ns: no significant; n = 3. **(G)** The mRNA expression of HSP60 was determined by qRT-PCR. *P < 0.05 vs. NC; n = 3. **(H,I)** Protein expression of TRPM4 was analyzed 48 h after transfection n = 6. **(J–M)** The mRNA expression of IL-6, IL-8, VCAM-1, and ICAM-1 were determined by qRT-PCR. *** <0.001, **P < 0.01 vs. Ctl; ^###^P < 0.001, ^##^P < 0.01, ^#^P < 0.05 vs. TNF-α; n = 3. **(N–Q)** The protein expression of VCAM-1 and ICAM-1 were determined by Western blot. **P < 0.01, *P < 0.05 vs. Ctl; ^#^P < 0.05 vs. TNF-α; n = 3.

Heat shock protein 60 (HSP60) plays a critical role in mediating pyroptosis and inflammatory responses by activating the NLRP3 inflammasome and the NF-κB signaling pathway (Ge et al., 2024). In the present study, TNF-α stimulation significantly upregulated both the protein and mRNA expression levels of HSP60 (Figures 4A–C), whereas the expression of HSP90 and HSP70 remained largely unchanged (Figures 4D–F), suggesting a specific involvement of HSP60 in the early phase of endothelial injury. Treatment with the TRPM4 inhibitor 9-Phe effectively suppressed the TNF-α-induced upregulation of HSP60 (Figures 4A–C) without exerting significant effects on HSP90 or HSP70 (Figures 4D–F). These observations indicate that TNF-α-induced endothelial inflammation is mediated, at least in part, through HSP60 upregulation, and that TRPM4 may function upstream of HSP60 in this process.

To further elucidate the functional role of HSP60 in endothelial inflammation, RNA interference was employed to knock down HSP60 expression in HUVECs. Transfection with HSP60-targeting siRNA achieved effective gene silencing, as confirmed by qRT-PCR; siHSP60-1 demonstrated the highest knockdown efficiency and was therefore used in subsequent experiments (Figure 4G). Notably, HSP60 knockdown did not significantly affect TRPM4 expression (Figures 4H,I), indicating that HSP60 acts downstream of TRPM4. We subsequently evaluated the effect of HSP60 silencing on the expression of key inflammatory mediators. Consistent with the effects of TRPM4 inhibition, HSP60 knockdown significantly attenuated the TNF-α-induced upregulation of IL-6, IL-8, VCAM-1 and ICAM-1 at both the mRNA and protein levels (Figures 4J–Q). These results further support the conclusion that HSP60 mediates the pro-inflammatory effects of TRPM4 signaling in endothelial cells.

### 3.5 HSP60 knockdown attenuates TNF-α-induced endothelial pyroptosis

**FIGURE 5:**
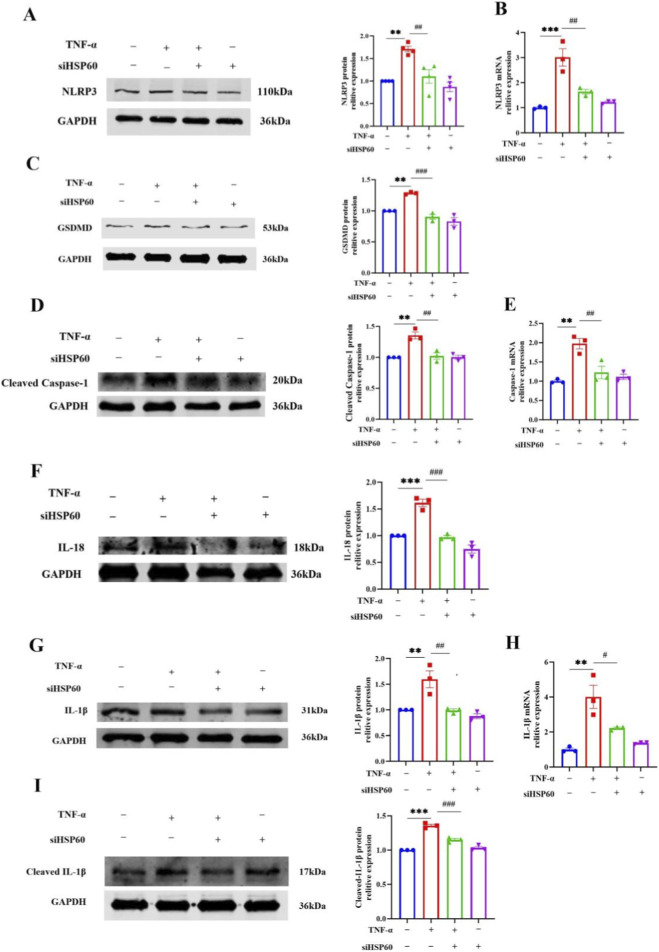
HSP60 knockdown attenuates TNF-α-induced pyroptosis in HUVECs. **(A)** Following transfection with siRNA targeting HSP60 (siHSP60) or negative control (NC), HUVECs were treated with 20 ng/mL of TNF-α for 12 h. The protein expressions of NLRP3 were determined by Western blot. **P < 0.01 vs. Ctl; ^##^P < 0.01 vs. TNF-α; n = 4. **(B)** The mRNA expression of NLRP3 were determined by qRT-PCR. ***P < 0.001, vs. Ctl; ^##^P < 0.01, vs. TNF-α; n = 3. **(C)** The protein expressions of GSDMD were determined by Western blot. **P < 0.01 vs. Ctl; ^###^P < 0.001 vs. TNF-α; n = 3. **(D,E)** The protein expressions of Cleaved Caspase-1 were determined by Western blot. The mRNA expression of Caspase-1 was determined by qRT-PCR. **P < 0.01 vs. Ctl; ^##^P < 0.01 vs. TNF-α; n = 3. **(F)** The protein expressions of IL-18 were determined by Western blot. ***P < 0.001 vs. Ctl; ^###^P < 0.001 vs. TNF-α; n = 3. **(G,H)** The protein expressions of IL-1β were determined by Western blot. The mRNA expression of IL-1β were determined by qRT-PCR. **P < 0.01 vs. Ctl; ^#^P < 0.05 vs. TNF-α; n = 3. **(I)** The protein expressions of Cleaved IL-1β were determined by Western blot. ***P < 0.001 vs. Ctl; ^###^P < 0.001 vs. TNF-α; n = 3.

Subsequently, we investigated the role of HSP60 in endothelial cell pyroptosis. The results showed that siRNA-mediated knockdown of HSP60 significantly inhibited TNF-α-induced upregulation of NLRP3, caspase-1 and IL-1β mRNA (Figures 5B,E,H). Consistently, the protein levels of NLRP3, GSDMD, cleaved caspase-1, IL-18, IL-1β and cleaved IL-1β were also substantially reduced following HSP60 silencing (Figures 5A–I). These findings demonstrate that HSP60 knockdown effectively attenuates the pyroptotic response in TNF-α-stimulated endothelial cells, supporting a critical role for HSP60 in mediating endothelial pyroptosis.

### 3.6 TRPM4 promotes NF-κB signaling activation by modulating the HSP60–IKKα/β interaction

**FIGURE 6:**
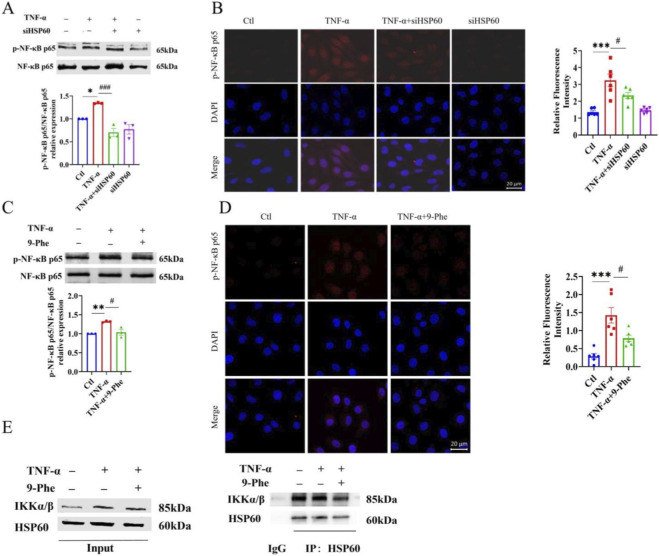
TRPM4 promotes NF-κB signaling by modulating the HSP60–IKKα/β interaction. **(A)** Following transfection with siHSP60, HUVECs were treated with 20 ng/mL TNF-α for 12 h. The ratio of phosphorylated NF-κB p65/NF-κB p65 was determined by Western blot analysis. *P < 0.05 vs. Ctl; ^###^P < 0.001 vs. TNF-α; n = 3. **(B)** Representative immunofluorescent images (left) and quantitative analysis (right) showing nuclear translocation of p-NF-κB p65. ***P < 0.001 vs. Ctl; ^#^P < 0.05 vs. TNF-α; n = 6. **(C)** HUVECs were treated with 20 ng/mL TNF-α and 1 μM 9-Phe for 12 h. The p-NF-κB p65/NF-κB p65 ratio was assessed by Western blot **P < 0.01 vs. Ctl; ^#^P < 0.05 vs. TNF-α; n = 3. **(D)** Representative immunofluorescent images (left) and quantification (right) of p-NF-κB p65 nuclear translocation. ***P < 0.001 vs. Ctl; ^#^P < 0.05 vs. TNF-α; n = 6. **(E)** Co-immunoprecipitation analysis of the interaction between HSP60 and IKKα/β in HUVECs treated as indicated; n = 3.

The expression of VCAM-1 and ICAM-1 is largely regulated by NF-κB signaling, as their gene promoters contain functional NF-κB binding sites. Inactive NF-κB bound to IκB in the cytoplasm is released upon IKK-mediated IκB phosphorylation and degradation, allowing nuclear translocation and target gene activation (Baeuerle, 1998). NF-κB p65 serves as a key transcriptional subunit, its activation is essential for TNF-α-induced inflammation in HUVECs (You et al., 2009). We examined NF-κB p65 phosphorylation and nuclear translocation in TNF-α-stimulated HUVECs following HSP60 knockdown or control treatment. HSP60 silencing significantly reduced TNF-α-induced p65 phosphorylation and nuclear accumulation (Figures 6A,B). These results indicate that HSP60 contributes to endothelial inflammation and pyroptosis, at least in part, through NF-κB activation.

We next examined whether TRPM4 influences NF-κB signaling. Inhibition of TRPM4 with 9-Phe significantly reduced TNF-α-induced phosphorylation of NF-κB p65 and attenuated its nuclear translocation (Figures 6C,D), indicating that TRPM4 acts upstream of NF-κB activation. Previous studies have suggested that cytoplasmic HSP60 can directly bind to IKKα/β and facilitate IKK/NF-κB signaling (Chun et al., 2010; Rufo et al., 2022). However, whether TRPM4 regulates this protein interaction remains to be determined. Using co-immunoprecipitation, we found that TNF-α enhanced the association between HSP60 and IKKα/β, whereas TRPM4 inhibition by 9-Phe attenuated this interaction (Figure 6E). These results demonstrate that TRPM4 promotes NF-κB pathway activation by enhancing the interaction between HSP60 and IKKα/β, thereby amplifying inflammatory and pyroptotic signaling in endothelial cells.

## 4 Discussion

Worldwide, atherosclerosis remains a leading cause of cardiovascular morbidity and mortality, underlying major disorders such as coronary artery disease, stroke and peripheral arterial disease (Engelen et al., 2022; Jebari-Benslaiman et al., 2022). The vascular endothelium is situated at the interface between the bloodstream and surrounding tissues, where it maintains homeostasis by regulating vascular tone, hemostasis, and inflammatory responses (Mallick and Duttaroy, 2022; Félétou, 2011; Baratchi et al., 2017). Endothelial inflammation and pyroptosis are key drivers of endothelial dysfunction and serve as critical initiators of early atherosclerotic lesions (Yu et al., 2024; Liu et al., 2024; Ju et al., 2022). In the context of atherosclerosis, inflammatory and immune pathways engage in complex crosstalk, which poses challenges for single-agent therapeutic strategies. Therefore, identifying novel pharmacological targets within these interconnected networks is essential to preserve endothelial integrity and limit the progression of atherosclerosis.

The TRPM4 channel, a Ca^2+^-activated but non-Ca^2+^-conducting cation channel, is a critical modulator of calcium signaling (Launay et al., 2002; Guo et al., 2017). However, its precise role in the integrated inflammatory and pyroptotic response remained elusive. In this study, we utilized tumor necrosis factor-α (TNF-α), a canonical pro-inflammatory cytokine (Xue et al., 2023; O'Carroll et al., 2015; Chen and Ma, 2019), to model endothelial activation. Our investigation revealed a pronounced, dose- and time-dependent upregulation of TRPM4 expression. Significantly, pharmacological inhibition of TRPM4 with 9-Phe potently suppressed the TNF-α-induced upregulation of key adhesion molecules (VCAM-1, ICAM-1) and cytokines (IL-6, IL-8) (Figures 2C–J), alongside critical mediators of pyroptosis (NLRP3, caspase-1, GSDMD) at both transcriptional and translational levels (Figures 3A–I). These findings unequivocally position TRPM4 as a key regulator of endothelial activation and a compelling therapeutic target.

In the present study, we observed that TRPM4 inhibition attenuated both the expression of adhesion molecules and chemokines and the activation of pyroptosis in TNF-α-stimulated HUVECs. Notably, pyroptosis not only results from inflammatory stress but also amplifies inflammation by promoting the release of IL-1β and IL-18, which in turn sustain the inflammatory response. Thus, the effects of TRPM4 on endothelial inflammation are mediated in part through its regulation of pyroptosis, indicating that TRPM4 acts upstream of a pro-inflammatory amplification loop.

Heat shock proteins are molecular chaperones that evolution has kept largely unchanged. Their job is to help cells survive when times get tough. Take HSP60: together with HSP10, it helps fold proteins inside mitochondria (Okamoto et al., 2017; Hauet-Broere et al., 2006; Grundtman et al., 2011). But that’s not all. HSP60 can also stir up inflammation, likely by tapping into Toll-like receptor signaling (Tian et al., 2013). When cells are under stress, it sometimes moves to the surface. And once there, it encourages leukocytes to stick around.

We know that calcium moving in and out of cells can influence how much HSP60 gets made. TRPM4, for its part, helps drive membrane depolarization and calcium entry. That connection got us thinking-maybe TRPM4 and HSP60 are functionally linked. Given that TRPM4 regulates calcium signaling, we next investigated whether TRPM4 modulates HSP60 expression in TNF-α-stimulated HUVECs. TNF-α stimulation significantly upregulated HSP60 expression, an effect that was suppressed by 9-Phe (Figures 4A–C). These results suggest the existence of a previously undescribed regulatory axis involving TRPM4 and HSP60. Notably, this regulation appeared specific to HSP60, as the expression levels of HSP90 and HSP70 remained unchanged (Figures 4D–G). Knockdown of HSP60 with siRNA attenuated inflammation and pyroptosis, whereas TRPM4 levels were unaffected (Figures 5B,C). These findings indicate that HSP60 acts downstream of TRPM4 signaling. This observation is consistent with clinical studies linking HSP60 to early atherosclerotic plaques (Xu L. et al., 2021; Rao et al., 2008), underscoring the relevance of the TRPM4-HSP60 axis in disease pathology.

The NF-κB signaling pathway plays a central role in endothelial inflammatory activation and is a well-established contributor to atherosclerosis (Li et al., 2022). We confirmed TNF-α-induced phosphorylation and nuclear translocation of NF-κB p65 and demonstrated that both HSP60 knockdown and TRPM4 inhibition abrogated this activation (Figures 6A–D). Building on previous reports that cytosolic HSP60 can bind IKKα/β to facilitate NF-κB signaling, our co-immunoprecipitation assays provided direct mechanistic evidence: TNF-α enhanced the interaction between HSP60 and IKKα/β, an effect that was substantially attenuated by TRPM4 inhibition (Figure 6E). These results support a coherent model in which TRPM4 activation facilitates the assembly of the HSP60-IKKα/β complex, thereby driving NF-κB activation and subsequent inflammatory responses.

**FIGURE 7:**
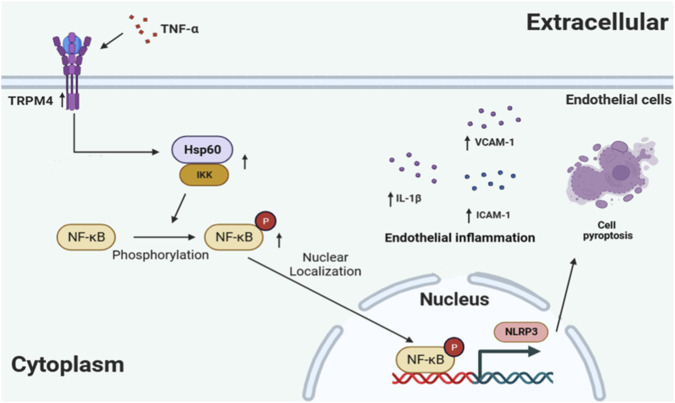
Proposed mechanism of the TRPM4-HSP60-NF-κB pathway in regulating inflammation and pyroptosis. TNF-α-induced endothelial inflammation upregulates TRPM4 expression. Pharmacological inhibition of TRPM4 alleviates TNF-α-induced endothelial inflammation and pyroptosis. TRPM4 facilitates the interaction between HSP60 and IKKα/β, leading to activation of the NF-κB signaling pathway. Modulation of the TRPM4-HSP60-NF-κB signaling axis represents a potential therapeutic strategy for ameliorating endothelial inflammation and pyroptosis in atherosclerosis.

This work identifiess a previously unrecognized signaling pathway-referred to as the TRPM4-HSP60-NF-κB axis. It appears to lie at the heart of how endothelial cells become inflamed and undergo pyroptosis. The finding points to a core regulatory mechanism. TRPM4 functions as an upstream controller. It strengthens how HSP60 binds with IKKα and IKKβ. From there, the NF-κB pathway gets switched on. Here is one particularly striking discovery: blocking TRPM4 alters how HSP60 is expressed. What emerges is a regulatory mechanism with multiple layers. The findings give us new reasons to consider targeting TRPM4 therapeutically. Rather than just blocking the channel itself, the idea now is to break up a key inflammatory signaling complex. This puts the TRPM4-HSP60-NF-κB axis in the spotlight as a promising multi-target opportunity. Our work makes a strong case for pushing ahead with new therapies aimed at TRPM4. If we can get it right, such strategies might 1 day help us treat atherosclerotic cardiovascular disease. A schematic diagram summarizing this proposed TRPM4-HSP60-NF-κB signaling pathway is presented in Figure 7.

## Data Availability

The raw data supporting the conclusions of this article will be made available by the authors, without undue reservation.
